# Methods of the Large-Scale Production of Extracellular Vesicles

**DOI:** 10.3390/ijms231810522

**Published:** 2022-09-10

**Authors:** Valeriia Syromiatnikova, Angelina Prokopeva, Marina Gomzikova

**Affiliations:** Laboratory of Intercellular Communication, Kazan Federal University, 420008 Kazan, Russia

**Keywords:** extracellular vesicles, microvesicles, exosomes, bioreactor, hypoxia, serum starvation, extrusion, ionophores, cytochalasin, sulfhydryl blocking agents

## Abstract

To date, extracellular vesicles (EVs) have been extensively investigated as potential substitutes for cell therapy. Research has suggested their ability to overcome serious risks associated with the application of these cells. Although, the translation of EVs into clinical practice is hampered by the lack of a cheap reasonable way to obtain a clinically relevant number of EVs, an available method for the large-scale production of EVs ensures vesicles’ integrity, preserves their biological activity, and ensures they are well reproducible, providing homogeneity of the product from batch to batch. In this review, advances in the development of methods to increase EVs production are discussed. The existing approaches can be divided into the following: (1) those based on increasing the production of natural EVs by creating and using high capacity “cell factories”, (2) those based on the induction of EVs secretion under various cell stressors, and (3) those based on cell fragmentation with the creation of biomimetic vesicles. The aim of this review is to stimulate the introduction of EVs into clinical practice and to draw attention to the development of new methods of EVs production on a large scale.

## 1. Introduction

The secretion of extracellular vesicles has been identified as a universal mode of intercellular communication between cells. According to the nomenclature of the International Society for Extracellular Vesicles (ISEV), the term “extracellular vesicles” (abbreviated as “EVs”) is used to refer to all particles surrounded by a double lipid layer and secreted by cells that cannot replicate [1]. EVs are a heterogeneous group of membrane structures, within which populations differ in terms of biogenesis and size: exosomes (50–150 nm)—vesicles of endosomal origin; microvesicles (50–500 nm to 1000)—vesicles budding from the plasma membrane; apoptotic bodies (>1000 nm)—vesicles formed by cell death [2]. In 2018, ISEV published updated guidelines for EVs research which aim to standardize experiments carried out by scientists in different laboratories [1]. EVs contain cellular components responsible for the unique biological function of the original (donor) cell, including various soluble proteins, lipids and genetic material, as well as organelles such as mitochondria [3]. The involvement of EVs in many physiological (immune response [4], blood clotting [5], aging [6], etc.) and pathological (cancer, infectious and endocrine diseases, neurodegenerative disorders [7]) processes has also been proven.

The biological effect of EVs on targeted cells is exerted both through the endocrine pathway affecting distant cells and the paracrine pathway affecting neighboring cells. EVs penetrate target cells apparently by various endocytic pathways such as phagocytosis, clathrin- and caveolin-mediated endocytosis, micropinocytosis, and membrane fusion [8]. Apart from EVs being studied in fundamental biology as carriers of cellular signals, there is ongoing research on their use as therapeutic tools for modulating physiological processes and drug delivery. EVs are considered potential substitutes for cell-based therapies, as they are able to overcome serious risks associated with the use of cells, such as malignant transformation, immune rejection, and embolization [9]. EVs secreted by mesenchymal stem cells (MSCs) are of particular interest, as numerous studies have confirmed that MSCs-derived EVs have broad regenerative potential in various human diseases [10]. EVs have shown renal- and cardio-protective activity [11,12] and effectiveness in skin lesions [13,14], diabetes [15], and myocardial infarction [16]. EVs can be used as drug delivery vehicles due to their ability to encapsulate various substances and penetrate physiological barriers. EVs have been used to deliver siRNA, microRNA, proteins, low-molecular-weight drugs, nanoparticles, and CRISPR/Cas9 to treat various diseases in animal models [17]. Thus, the development of therapeutic tools based on EVs is a promising field in regenerative medicine.

Despite this, the use of EVs in clinical practice is currently difficult for several reasons. First, cells secrete a number of EVs insufficient for clinical translation [18]. While the used dose of exosomes in most studies is approximately 10–100 µg exosomal protein/mouse, the yield of EVs is usually less than 1 µg of exosomal proteins per 1 mL of culture medium [19]. Second, current methods of isolating EVs are either time-consuming or expensive. Third, there is no clinically feasible method for the scalable production of EVs with reproducible properties. Lastly, no production protocol has been developed to comply with Good Manufacturing Practice (GMP) and control the “quality” of the EVs produced.

**Figure 1:**
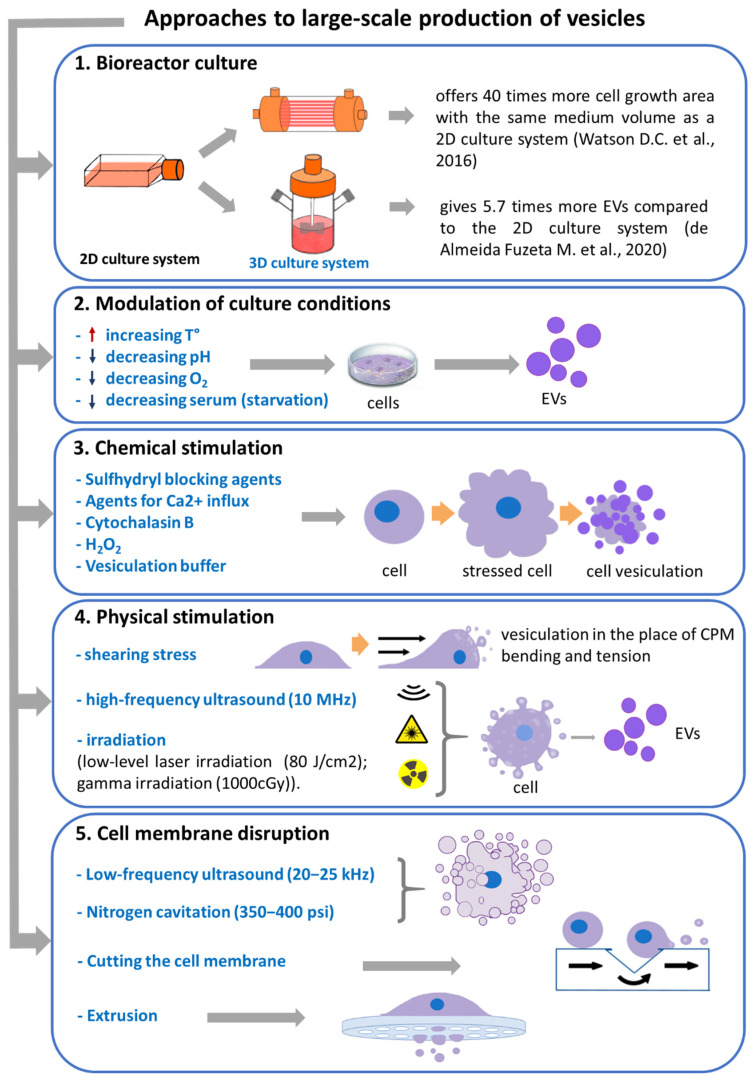
Approaches to the large-scale production of EVs and biomimetic vesicles. CPM—cytoplasmic membrane.

Thus, to create pharmaceutical products based on EVs, it is necessary to overcome the above-mentioned obstacles and develop a scalable method of producing EVs in which a clinically relevant number of EVs with reproducible characteristics can be obtained. This review focuses on recent advances in the development of methods for the large-scale production of EVs (Figure 1, Supplementary Material) and discusses the advantages and disadvantages of these methods.

## 2. Bioreactor Culture

Traditionally, the two-dimensional flask culture method has been used for the cultivation of adherent cell cultures. Multilayer culture systems have also been created to scale up culturing. Andriolo G. et al. used multilayer culture vessels to generate EVs from human cardiac progenitor cells (Exo-CPC) on a large scale in 2D culture and in compliance with GMP standards [20]. The authors cultured the cells in xeno-free conditions, harvested up to 8 L of the conditioned medium, and isolated Exo-CPC by tangential flow filtration (TFF). The authors developed and performed quality control tests involving the evaluation of the safety and identity of both cardiac progenitor cells (CPC) as a cell source and Exo-CPC as an end product. Although the cells showed lower doubling times in xeno-free conditions than those observed in research-grade conditions, the developed GMP production assured a high exosome yield (>10^13^ particles) and the stable removal (≥97%) of protein impurities [20].

Kang H. et al. developed a culture plate bioreactor with an attached peristaltic pump and gas delivery system in which cells were shear-stressed by an introduced medium, inducing higher EVs production than that in static cell culture. The authors showed that the bioreactor produced about seven times more EVs per cell than static conditions in 1 day [21].

Another example of a bioreactor flask is a modified flask consisting of two chambers separated by a semi-permeable membrane with a molecular weight cut-off of 10 kDa, which separates the chamber from the cells and from the growth medium. This eliminated the risk of contamination of the product with serum EVs. The T-175 flask-sized bioreactor system is static and does not require pumps with actuators. The further isolation of EVs from the conditioned medium is performed by ultracentrifugation [22].

The two-dimensional culture method is less physiological favorable for the cells, and the culture growth in flasks is limited by the surface area of the culture plastic. This results in a slow culture growth rate and a low yield of EVs. The solution to the problem of the low yield of EVs in 2D cell culture systems is the use of microcarriers, scaffolds, and hollow capillaries in 3D culture systems. To scale up the production of EVs, various bioreactor systems are being used to optimize nutrient consumption and increase the surface area for cell growth. Modified bioreactor systems are being developed that separate the culture medium chamber and the cell chamber. This separates secreted EVs from serum contaminants. In addition, bioreactors are supplemented with shear stress inducers to stimulate the release of vesicles.

Haraszti R.A. et al. cultured umbilical cord MSCs in a bioreactor combined with microcarriers to increase the EVs yield and then isolated EVs using either differential ultracentrifugation (UC) or TFF. The authors estimated that 1.4 × 10^4^ particles/cell were obtained in a 3D culture using UC and 1 × 10^5^ particles/cell were obtained using TFF, whereas the number of particles obtained per cell was 7 × 10^2^ and 2 × 10^4^ in a 2D culture, respectively [23]. de Almeida Fuzeta M. et al. developed a scalable production of EVs from MSCs on microcarriers in a Vertical-Wheel bioreactor (VWBR). Compared to conventional 2D culturing systems, the VWBR system produced 5.7 times more EVs in the same volume of the nutrient medium [24].

One of the most capacious bioreactors that provides the largest growth surface area to a given volume of a medium is the hollow fiber bioreactor. This bioreactor is a 3D culturing system consisting of hollow fibers and small semi-permeable membranes with a molecular weight cut-off, which are bound together in a tubular cartridge. The cells are attached to the outside of the hollow fibers, within which the culture medium is continuously pumped, allowing for nutrient and gas influx without the contamination of EVs from serum [25]. Watson D.C. et al. showed that culturing HEK293 in a FiberCell System hollow fiber bioreactor gives 40 times more EVs per volume of a conditioned medium compared to 2D cell culture. Thus, approximately 53 large (175 cm^2^) flasks and 800 mL of the conditioned medium are required to match the single daily collection of the FiberCell bioreactor (20 mL) [26].

Bioreactors were equipped with shear stress inducers to stimulate cells to increase EVs production. Continuous medium perfusion (laminar flow) or turbulent flow act as shear stress inducers. Patel D.B. et al. compared the efficiency of EVs production in 2D static culture, 3D static culture (without medium perfusion), and 3D dynamic culture with medium perfusion (which gives a small shear stress). The authors found a 100-fold increase in EVs production in 3D dynamic culture with perfusion compared to 3D static culture and 2D static culture on day 1 and a 10,000-fold increase in EVs production on day 3 [27].

Guo S. et al. also used the laminar flow of medium perfusion to induce EVs production and demonstrated that flow stimulation increases the yield of EVs by 3.4–37 times depending on the cell type (dental pulp and adipose tissue stem cells) and flow rate in comparison with static (without laminar flow) conditions [28].

Shearing induced by laminar or turbulent flow probably induces the elongation of the lipid membrane. This causes the membrane to be thermodynamically stable, eventually leading to the budding of small vesicles [29,30]. Depending on the degree and intensity of shear stress on cells, membrane fragmentation with the leakage of intracellular contents (e.g., proteins, RNA) and the spontaneous self-assembly of EV-like vesicles may occur. The resulting EV-like vesicles are probably heterogeneous, with different molecular weights [30].

Existing bioreactor systems are being upgraded to increase the production of EVs or to separate EVs from impurities in the nutrient medium. According to the Espacenet database (accessed on 30 April 2022, Table 1), bioreactor systems based on culture bottles (CN110872562 (A)) or stirred bioreactors (WO2020136362 (A1)) were developed for producing EVs, the basic idea of which is to embed an Evs separation module for extracellular vesicle separation. Such an Evs separation module is typically a tank with sequentially arranged filter elements to filter the producer cells (with pore sizes of 2 μm), to filter the cellular debris (with pore sizes of 1 μm), and to extract EVs with a diameter of 0.1–1 μm by filtering the medium out (with pore sizes of 0.1 μm) (CN110872562 (A); WO2020136362 (A1)).

Despite the advantage of bioreactor systems in terms of the efficient use of space and the conservation of media, the cell source for the production of EVs has the following limitations: (1) cell aging; (2) the cessation of adherent cell division when reaching the monolayer; (3) the spontaneous differentiation of undifferentiated cells (e.g., MSCs), resulting in the release of a mixture of EVs with unpredictable properties [31]. However, individual studies did report the possibility of culturing human bone marrow MSCs in a bioreactor for at least 28 days with the preservation of viability (>85%), functionality, and immunophenotypic characteristics [32]. A possible solution to overcome the above-mentioned limitations could be the immortalization of cell lines, which has been shown not to affect the quality and quantity of EVs produced [33], as well as the quality control of the cell source and the resulting product.

Quality control standards of EVs should include a vesicle production protocol that would at least evaluate the following parameters: morphology, integrity, homogeneous size, the absence of EVs aggregates, and the absence of contaminants in the form of xenogenic components of the nutrient medium on delivery. Moreover, each batch of produced EVs must undergo a biological activity evaluation, which should include an assessment of the molecular composition, a comparative analysis for compliance with reference values, and an assessment of activity using an appropriate test system.

Most of the data on the biological activity of EVs were obtained using 2D cell cultures. It should be noted that it is required to provide data supporting that the composition and biological activity of EVs obtained from 3D cell cultures do not differ from those for EVs obtained from 2D cell cultures. To date, few studies have demonstrated that EVs derived from a 3D cell culture showed an increased regenerative potential in cartilage [34], kidney [35], skin [27], and heart [36] treatment compared to EVs derived from a 2D cell culture [37].

## 3. Cell Stimulation

EVs are released spontaneously into the extracellular medium from the cells. Changing the cultivation conditions and applying chemical or physical inducers, however, stimulates the production of more EVs. Methods to increase EVs production are being developed based on stress stimuli.

### 3.1. Modulation of Cultivation Conditions

**Serum starvation.** Fetal bovine serum (FBS) is usually used as a source of nutrients and growth factors in the culture medium. Restricting cell nutrients by reducing or excluding serum from the medium (use of a serum-free medium) leads to an increased production of EVs. Sun L. et al. showed that serum deprivation stimulated the production of EVs from the human myeloma cells RPMI 8226, U266, and KM3 by 2.5, 4.3, and 3.8 times, respectively [38]. Contrary results were obtained in a study by Saludas L. et al. The authors found that serum starvation led to a sharp decrease in EVs secretion in a culture of cardiac progenitor cells [9]. In addition, it was shown that the addition of serum-depleted EVs to the medium increases the yield of EVs compared with the use of a serum-free medium [39]. This contradiction is probably due to the fact that, even in the EVs-depleted serum, there are protein complexes and EVs, which could be taken into account in the estimation of the EVs yield. Shelke G.V. et al. showed that an 18 h UC protocol eliminates approximately 95% of the FBS EVs [40], which is supported by our data.

**Changing pH.** Another exogenous factor stimulating the production of EVs by cells is the change in proton concentration. The results of Parolini I. et al. showed that melanoma cells secreted more exosomes in an acidic medium than they did in a buffer medium [41]. However, it remains unclear whether the pH-dependent secretion of EVs is only characteristic of pH-sensitive cells such as cancer cells.

**Temperature shift**. It has been reported that thermal stress is another way to stimulate cells to secrete more EVs. When human T cell leukemia Jurkat and B cell leukemia/lymphoma Raji cell lines were subjected to thermal stress at 40 °C for 1 h, the production of EVs was increased by 3- and 22-fold, respectively [42,43]. In a study by Chen T. et al., the incubation of 3LL lung cancer cells at 42 °C for 1 h, along with a 4 h recovery period, resulted in a ~2.5~3.5-fold increase in exosome production as compared to that obtained from a 4 h culture supernatant of 3LL cells without hyperthermia [44]. Otsuka K. et al. identified the molecular mechanism of temperature stimulation and showed that, in breast cancer, the thermostable low-density lipoprotein receptor (LDLR) plays a role in the temperature-dependent increase in EVs secretion [45].

**Hypoxia.** The secretion and composition of EVs are closely related to the oxygen content in the cell microenvironment [46]. In a study conducted by King H.W. et al., moderate and severe hypoxia (1% and 0.1% O_2_, respectively) promoted the significant release of EVs by breast cancer cells [47]. Similar results were observed when ovarian cancer cells were exposed to hypoxia—EVs production was increased by the activation of Rab27a, the suppression of Rab7, LAMP1/2, and NEU-1, and by the stimulation of a more secretory lysosomal phenotype [48]. However, there is evidence that hypoxia has a significant effect on the composition of EVs. For example, hypoxia increased the relative expression of a large number of bioactive factors involved in angiogenesis in the composition of EVs of endothelial progenitor cells [49].

Most of the studies mentioned above have been performed on cancer cell lines, which are characterized by an increased capacity for EVs secretion [50]. Therefore, the results obtained require the confirmation and optimization on non-cancerous cells. It is important to note that the application of stressful cultivation conditions may affect the composition, characteristics, and functions of produced EVs. In addition, stress can increase the contamination of apoptotic bodies due to cell death, which can have adverse effects and lead to errors in determining the yield of EVs [19]. Therefore, it is necessary to assess not only the yield of the obtained EVs but also their quality and activity.

**Additives in a culture medium.** According to the Espacenet database (accessed on 30 April 2022, Table 1), adding sodium pyruvate, insulin, transferrin, and sodium selenite to the MSCs culture medium stimulates EVs production (KR102284517 (B1)). In order to stimulate the production of EVs by cells, it has been suggested to add the following components to the medium: lipopolysaccharide and nigericin (CN112011499 (A)), plant ceramide (US2021177778 (A1)), factor complex—EGF, TGF, coenzyme Q10, potassium salt, fructose sodium diphosphate, and histamine dihydrochloride (CN112920996 (A)), factor complex—L-ascorbic acid or salt thereof, selenium or salt thereof, NaHCO_3_, and insulin (CN112920991 (A)), TNF-α and interferon-γ (WO2021020726 (A1)), platelet lysate (KR102209937 (B1)), and VCAM-1 (CN110195038 (A)).

### 3.2. Application of Chemical and Physical Inducers

In this section, we have reviewed the methods of increased vesicles production. Due to the induced nature of the obtaining vesicles, the authors underline their difference from naturally formed EVs using such terms as “induced EVs”, “membrane vesicles”, or “vesicles”. We believe that the main difference between the vesicles described below and natural EVs is the violation of the mechanism of the specific sorting of EVs composition during formation.

#### 3.2.1. Chemical Induction

The application of chemical inducers is a promising way to introduce the clinical application of EVs, which allows for the production of a homogeneous fraction of EVs in a short period of time. The action of chemical inducers targets the cell cortex, mainly actin. The loss of membrane-cortex adhesion or induced cortex weakening and cytoplasmic pressure lead to herniations of the membrane that grow into spherical protrusions called blebs [51]. Thus, EVs obtained by the blebbing of plasmalemma retain the membrane components and cytoplasmic contents of the cell, making them a promising tool of therapy or a means of drug delivery.

**Sulfhydryl-blocking agents.** The blebbing of the cell membrane is observed when the membrane is exposed to a sulfhydryl-blocking reagent. This effect was first described by Scott R.E. et al., who reported that the exposure of 3T3 and SV3T3 mice embryonic cells to a sulfhydryl-blocking agent, formaldehyde, along with a disulfide-reducing agent, dithiothreitol (DTT), induced the formation and release of plasma membrane vesicles [52]. However, sulfhydryl blockade often results in the formation of giant membrane vesicles with a high heterologous size distribution [53].

Ingato D. et al. optimized this sulfhydryl-blocking method by adding paraformaldehyde to DTT to obtain nanovesicles with a high homogeneity [53,54]. Using this approach, more than an order of magnitude more nanovesicles were obtained after 2 h of vesiculation than naturally occurring EVs collected over 48 h, directly measured by the protein amount. However, these active chemicals may introduce artifacts in the experimental results: DTT is a reducing agent, and formaldehyde may induce membrane protein cross-linking, thereby affecting the measured level of interactions [55].

**Ca^2+^.** The biogenesis of plasma membrane-derived EVs is induced by an increase in intracellular calcium, which leads to changes in the membrane lipid distribution and the disruption of cytoskeleton integrity [56]. The application of monensin ionophore stimulated the release of EVs of the human erythroleukemia cell line K562 in a calcium-dependent manner [57]. Ca^2+^ ionophore treatment has also been used to increase EVs secretion by oligodendroglial cells [58], dendritic cells [59], and mast cells [60]. In a recent study, biomaterials (calcium phosphate nanoparticles) that release Ca^2+^ ions were introduced into murine macrophage-like RAW264.7 cells and human monocyte-like THP-1 cells, resulting in a significant increase in EVs secretion by more than two times [61,62]. However, ionophores exhibit cytotoxicity and reduced cell viability [63].

**Cytochalasins.** Cytochalasin is a fungal metabolite which inhibits the formation of actin filaments via binding to the barbed ends of actin filaments, which inhibits both the polymerization and depolymerization of actin subunits at this end [64,65,66]. This causes the disruption of the cytoskeleton integrity, which is a necessary condition for the detachment of EVs from the cytoplasmic membrane. Pick et al. demonstrated that the active agitation of cytochalasin B-treated cells stimulated the production of membrane vesicles [67]. Nair A. et al. reported that cytochalasin B at a concentration of 10 μg/mL promotes the production of EVs by cells, producing approximately three times more particles than natural EVs secretion [68]. It was previously demonstrated that cytochalasin B-induced membrane vesicles contain the cytoplasmic content of parental cells, retain an immunophenotype, and exhibit angiogenic activity [69,70]. Cytochalasin B-induced membrane vesicles of MSCs exhibit immunosuppressive activity in vitro [71] and in vivo [72].

**H_2_O_2_.** Hedlund M. et al. found that oxidative stress induced by a high concentration of hydrogen peroxide (50–100 µM) stimulated a 15-fold and 32-fold increase in the release of EVs by tumor cells compared to normal cultivation conditions [42,43].

**Vesiculation buffer.** The process of cell membrane vesiculation can also be triggered by the difference between intracellular and extracellular osmotic pressure. Cohen S. et al. developed a method of obtaining vesicles from a highly adherent line of A431 cancer cells by osmotic shock [73]. Later, Del Piccolo N. et al. applied this method to Chinese hamster ovary (CHO) cells [55]. The method involves hypotonic washing followed by incubation in an osmotic (vesiculating) buffer. The osmotic buffer does not rupture the cells, but it does stress them, so they produce vesicles.

**Hyaluronic acid.** It was found that the cultivation of stem cells in the presence of hyaluronic acid increased stem cell proliferation by about 364%, supported their stemness, and improved exosome production efficiency by 5.2 times (patent KR102123268, Table 1).

**Drugs.** Clinically approved medicines such as pioglitazone, metformin, and 5-aminoimidazole-4-carboxamide ribonucleotide (AICAR) were shown to be able to stimulate EVs release by 161%, 122%, and 148%, respectively (patent WO2019107939, Table 1). It was found that the simultaneous inhibition of glycolysis (using sodium iodoacetate) and oxidative phosphorylation (using 2,4-dinitrophenol) triggers a 3- to 24-fold increase in the secretion of EVs (patent WO2021040999, Table 1). Drugs induced 2′,3′-cAMP accumulation, although the mechanism by which this increases exosome release remains unknown (patent WO2021040999, Table 1, [74]).

#### 3.2.2. Physical Stimulation

**Shear stress.** Miyazaki Y. et al. showed that increasing the duration of shear stress proportionally increases the formation of EVs from the plasma membrane of platelets [75]. Morrell A.E. et al. demonstrated that the stimulation of fluid shear flow (35 dynes/cm^2^) increases the production of EVs in osteocyte-like MLO-Y4 cells by approximately 20 times compared to the control [76]. As we described above, shear stress is widely used in bioreactor systems to increase EVs budding by cells (patent US2021189329, Table 1).

**Acoustic treatment.** Ultrasonic treatment is a widely used tool for the preparation of liposomes [77]; later, it also began to be used to increase EVs production. In a study by Zhao Z. et al., the exposure of ovarian cancer cells (A2780) to low-intensity ultrasound (0.5 W/cm^2^) for 60 min significantly promoted exosome secretion without significant changes in morphology, size, and distribution in vivo [78]. Ambattu L.A. et al. found that the exposure of cells to high-frequency acoustic radiation stimulated exosome generation without affecting cell viability. The authors state that, within seven cycles, which corresponds to a total treatment duration of 280 min, an 8–10-fold increase in the number of exosomes can be obtained [79]. In addition, ultrasound exposure is used to split cells into vesicles, but we do not classify this method as a way to “stimulate” EVs production. Mechanical methods that lead to complete cell cleavage will be discussed below as methods of producing biomimetic vesicles.

**Irradiation.** Human endothelial cells were treated with low-level laser irradiation (LLLI) at a power density of 80 J/cm^2^. According to the data from a TEM analysis, an augmented secretion of exosomes was found in HUVECs after irradiation with 80 J/cm^2^ LLLI in comparison with the control and 2 J/cm^2^. But higher doses, including 60 and 80 J/cm^2^, yielded an approximately 1.06- and 1.2-fold reduction in cell viability after 48 h [80]. In a study by Wysoczynski M. and Ratajczak M.Z., the murine (LL-2) and human (A549) lung cancer cell lines were subjected to gamma-irradiation (1000 cGy). LL-2 and A549 cells secreted 4-fold more MV 12–48 h after gamma irradiation compared with controls, while the doses used had no effect on the viability of LL-2 and A549 cells, according to the authors [81].

**Microgravity and zero gravity.** It was found that the culture conditions under microgravity (1/1000 G) increased the number of extracellular vesicles secreted from mesenchymal stem cells by 10% (patent WO2021162114, Table 1).

**Magnetic nanoparticles and magnetic force.** Applying positively charged polymer-magnetic nanoparticle to mesenchymal stem cells and 0.3–1 T magnetic forces induced the generation of EVs (patent WO2021086139, Table 1). However, the authors did not specify the exact increase in EVs generation.

The quality control measures of induced EVs are broader than those of natural EVs, and, besides the evaluation of EVs morphology, integrity, homogeneous size, the absence of EVs aggregates and xenogenic components, and the assessment of biological activity, should include the following tests: a low level or absence of chemical contaminants and cellular components in free form (nuclear DNA, organelles, proteins) which might have a negative influence on recipient cells. Each batch of induced EVs, similar to natural EVs, must undergo a biological activity evaluation and additional tests for toxicity and immunogenicity.

## 4. Production of Biomimetic Vesicles

In addition to increasing the yield of natural EVs, methods are being developed for the production of EV-like biomimetic vesicles, which involves the disruption of the cell membrane followed by the self-assembly of membrane vesicles.

**Homogenization by ultrasound.** Wang L. et al. developed an approach that, by homogenizing cells with ultrasound, helps to obtain a vesicle yield 20-fold higher and about 100-fold faster than the natural secretion of EVs. EVs obtained in this way had similar morphologies, size distributions, and typical protein markers to natural EVs and promoted wound healing in mice [82].

**Nitrogen cavitation.** Another approach was employed by Gao J. et al. to fabricate nanovesicles using nitrogen cavitation, which rapidly disrupts activated neutrophils to form nanovesicles from the cell membrane [83]. The yield of nanovesicles obtained in this way was 16 times higher compared to that of naturally secreted EVs.

**Extrusion.** Cell extrusion was another rapid method for the preparation of EV-like vesicles, where cells are pushed through porous filters of different sizes by hand or by using centrifugal force with a uniform application of pressure [30]. When a cell passes through the filter pores, the surface of the plasma membrane is stretched by the frictional force on the filter surface, while tension causes the plasma membrane to elongate [84]. When the yield point is reached, the lipid bilayer undergoes plastic deformation and rupture, followed by the rupture of linear fragments of the lipid double layer, which reassemble rapidly and spontaneously into spherical vesicles [30,85]. Jang S.C. et al. first developed bioinspired exosome-like nanovesicles by serially disrupting monoblasts and macrophages by passing through 10 μm, 5 μm, and 1 μm filters followed by UC in a density gradient. The resulting nanovesicles of cellular origin had similar characteristics to exosomes, but their production was 100-fold higher [86]. Jo W. et al. developed a device using centrifugal force and a micro-sized polycarbonate filter to generate EVs. When the centrifuge operates, nanovesicles are directly created from cells that elongate while passing through hydrophilic micro-size pores. The quantity of nanovesicles produced using the device is 250 times the quantity of naturally secreted exosomes [87]. The authors also propose a novel and efficient method to generate artificial nanovesicles via cells flowing through slits in hydrophilic microchannels. The authors suppose that nanovesicles are generated possibly due to abrupt pressure change, and their elongated shape is caused by shear stress [29].

**Cutting the cell membrane.** Yoon J. et al. developed a microfluidic system for generating nanovesicles. Living cells that have passed through microchannels were cut by microfabricated 500 nm-thick silicon nitride blades. Afterward, the fragments of the plasma membrane, cut from the cells, were self-assembled into spherical nanovesicles that were 100–300 nm in diameter. The average amount of protein and the number of nanovesicles generated by 1 million embryonic stem cells were measured as ∼20 µg and ∼150 × 10^8^ particles, respectively [88].

The main disadvantages of the above-mentioned strategies for producing EVs-like biomimetic particles are the inability to recycle cells, the loss of cytoplasmic content, the release of enzymes that degrade bioactive molecules, and the risk of the contamination of vesicle preparation with nuclear components. Since the aforementioned methods can alter the integrity of the cell membrane and impair the original functionality, biological activity, and biodistribution, the quality control of EV-like biomimetic vesicles must be extremely strict. In our opinion, such quality control as the total profiling of proteins, lipids, and nucleic acids to control biomimetic vesicles composition and the test of any functional alteration of biomolecules and stability should be additionally included in the aforementioned tests. Each batch of natural and induced EVs biomimetic vesicles must undergo a biological activity evaluation and additionally be tested for toxicity and immunogenicity.

## 5. Conclusions

EVs are a promising therapeutic tool for modulating physiological processes and drug delivery. However, to achieve a pronounced clinical effect, large doses of EVs are required, significantly exceeding the yield of natural vesicles secreted by cells. Therefore, a robust, cost-effective, and scalable method to produce clinical-quality EVs is required.

We believe that each of the reviewed methods of large-scale EVs production has its own advantages. The advantage of natural EVs obtained using bioreactor systems is their natural production and ability to retain the therapeutic potential of parental cells. The advantage of methods applied during induced EVs production is the higher EVs yield. To additionally scale up the production procedure, these methods can be combined with other methods for increased EVs production—for example, with bioreactors. The methods of biomimetic vesicles production require lower labor and time costs and produce EVs with a homogeneous size, high yield, and homogeneous composition. All of the described methods of EVs mass-production are promising and have the potential to be adapted and implemented in large-scale production. Apparently, the introduction of new EVs-based therapeutics into clinical practice will follow a conservative path if there is a scaled-up cell cultivation with the optimization of EVs isolation and purification procedures. However, once the first FDA-approved EV-based therapeutics become available, the demand for the methods described above to increase EV yield is likely to increase significantly. In conclusion, it should be noted that the yield and purity of EVs-based therapeutics depend not only on the production methods but also on the effectiveness of the EVs isolation method. To date, ultracentrifugation remains the most commonly used method for EVs isolation. However, ultracentrifugation leads to a low yield of EVs and contamination with medium proteins. Therefore, microfiltration, size exclusion chromatography, and immunoisolation are becoming increasingly preferred methods for effective EVs isolation.

## Figures and Tables

**Table 1:** Overview of patents related to methods of membrane vesicles production.

Publication Info	Priority Date	Production Method	Results
Bioreactor systems			
CN110872562 (A)	2019-11-28	Multiple modules device based on a stirred bioreactor where special condition—starvation is applied. EVs separation using filters.	Cal27 cell-derived EVs have a diameter distribution in the range of 100–1000 nm.
WO2020136362 (A1)	2018-12-28	Fluidic system for producing EVs, in which the suspension cell culture turbulent flow of ≤50 µm Kolmogorov length is applied.	Average diameter of EVs is between 50 and 500 nm—preferably between 100 and 110 nm.
KR20190010490 (A) KR102176500 (B1)	2017-07-21	Method for producing EVs using a perfusion bioreactor. Shear stress 0–0.5 dyne/cm^2^ and media flow ≤5 mL/min.	Size of EVs is 145 nm, on average. The number of EVs produced in the bioreactor is about six times greater than the number EVs secreted from the cell culture dish (control).
CN110564682 (A) CN110564682 (B)	2019-09-30	A method for the large-scale production of human adipose-derived MSCs exosomes which includes cell cultivation on microcarriers in a stirred bioreactor and stimulation using a fibroblast-conditioned medium and a hypoxic environment (3–5% O_2_).	Exosomes are round-like vesicles with a complete membranous structure, and the diameters are concentrated in 60–100 nm.
Culture conditions			
KR102284517 (B1)	2021-01-15	The medium for promoting the production of exosomes and/or EVs derived from human MSCs, which contains sodium pyruvate, insulin, transferrin, and sodium selenite.	The number of EVs is increased by 2.122% compared to the negative control.
CN112011499 (A)	2020-09-10	A method for preparing EVs which includes establishing a cell pyroptosis model through co-culture cells with lipopolysaccharide and nigericin.	EVs with a diameter of 2–5 μm.
US2021177778 (A1)	2017-03-14	An agent for promoting exosome production which contains a ceramide (6–26 carbon atoms) as an active ingredient.	The number of exosome particles correlate with the increase in the concentration of ceramide.
CN112920996 (A)	2021-04-23	The exosome secretion medium for umbilical cord mesenchymal stem cells, which contains EGF, TGF, coenzyme Q10, potassium salt, fructose sodium diphosphate, and histamine dihydrochloride.	The ratio of the number of cells to the number of secreted exosomes is about 1:50 to 1:80 instead of 1:5.
CN112920991 (A)	2020-12-31	The exosome secretion-inducing medium, which contains a basic culture medium, DMEM/F12, and additives: L-ascorbic acid or salt thereof, selenium or salt thereof, NaHCO_3_, and insulin.	The yield of exosomes obtained by inducing iPSC culture using the developed medium is 59 times higher than that obtained by inducing MSC culture using an MSC medium.
WO2021020726 (A1)	2019-07-30	A method for producing an exosome comprising the step of culturing an animal-derived cell in a medium containing TNF-α and interferon-γ (concentration of about 1–100 ng/mL).	Exosome productivity is approximately 2–3 times higher than that of the untreated control group.
KR102209937 (B1)	2020-06-22	Production method of exosomes for improving the productivity base on a serum-free medium, to which platelet lysate is added (0.01–20%).	The productivity of the number of exosome particles per ml of culture medium is approximately two or more times higher that without treatment.
CN110195038 (A)	2019-05-08	The application of VCAM1 to increase the output of exosomes. Cell culture medium contains 100 mL DMEM/F12 medium + 2 mL non-essential amino acids + 2 mL L-glutamine + 2 μg VCAM1 reagent.	The production of exosomes is significantly increased by more than 10 times.
CN107475187 (A)	2017-09-05	A culture solution for obtaining a large number of umbilical cord MSCs exosomes which contains recombinant interferon gamma (45–55 μg/L), serum substitute, penicillin, and streptomycin.	The addition of recombinant interferon γ increases the number of exosomes in the culture medium by more than 40%.
Chemical induction			
WO2021040999 (A1)	2019-08-27	Methods for improving the stimulation of the secretion of exosomes with the treatment of sodium iodoacetate (glycolysis inhibitor) plus 2,4-dinitrophenol (oxidative phosphorylation inhibitor), up to 10 mM each.	A 3-fold increase of exosome secretion after 6 h, an almost 6-fold increase after 12 h, and a >10-fold increase for cellular treatments longer 48 h.
KR102123268 (B1)	2019-06-11	Composition for promoting the generation of stem cell-derived exosomes containing hyaluronic acid (1–1000 ug/mL).	Compared to MSCs not treated with the substance, about 5.2 times more exosomes are produced in cells pretreated with hyaluronic acid.
WO2019107939 (A1)	2017-11-29	Composition for promoting the generation of stem cell-derived exosomes comprising one or more substances selected from the group consisting of 4 μM pioglitazone, 4 mM metformin, and 100 μM 5-aminoimidazole-4-carboxamide ribonucleotide.	Exosomes production increases by 22–61%.
Physical induction			
US2021189329 (A1)	2019-06-26	Methods and systems for the enhanced production and/or secretion of extracellular vesicles from a three-dimensional porous scaffold with a population of stem cells. Shear stress stimulations of about 0.5 dyne/cm^2^.	The EVs mean size among all groups (0.5 mL/min: 172.9 ± 3.048 nm; 1.0 mL/min: 203.2 ± 13.05 nm; 3D: 179.6 ± 8702 nm; 2D: 161.2 ± 5.502 nm).
WO2020071662 (A1)	2018-10-02	Method for the preparation of exosomes comprising the steps of: providing direct (0.1–3 W/cm^2^, 20 kHz–20 MHz) or indirect (1–20 W/cm^2^, 20 kHz–20 MHz) ultrasonic stimuli for cells (duration of 0.1 s to 20 min).	The exosomes diameter is 50–200 nm. The exosomes number using this method is four times greater than the number of exosomes secreted from the same number of cells without stimulation.
WO2021162114 (A1)	2020-02-14	A method for preparing EVs which comprises culturing cells in a microgravity or weightless environment of less than 1 G.	The number of EVs is 6.9 × 10^8^ particles/mL in normal gravity (1 G), whereas it was 7.8 × 10^8^ particles/mL in microgravity (1/1000 G).
WO2021086139 (A1)	2019-10-30	A method for promoting the generation of stem cell-derived exosomes using polymer-magnetic nanoparticle clusters (with a positive charge) and applying a magnetic force (0.1–2 T).	The generated exosomes have a size of 91–169 nm.
Biomimetic vesicles			
CN111235108 (A)	2020-02-19	The production method of the membrane nanovesicles, which comprises sequentially and repeatedly extruding a single-cell suspension to pass through polycarbonate membranes with different pore diameters (1, 5, and 10 µm).	The particle size of the vesicles is 100–200 nm. 5 × 10^6^ cells can produce 1.16 × 10^10^ cell membrane nanovesicles, and a single cell can produce 7.730 membrane nanovesicles
Genetic modification of cells			
US2021113662 (A1)	2018-04-18	Stem cells overexpression of CAMKK1 (calcium/calmodulin-dependent protein kinase kinase 1) enhances exosome release into the conditioned media.	The number of exosomes (3.76 × 10^9^ ± 2.2 particles/mL, *p* < 0.05) is higher than that of the control group (2.7 × 10^9^ ± 4.9 particles/mL).

The patent search was carried out at https://ru.espacenet.com/ (accessed on 30 April 2022).The search query included the terms “production vesicles” and “production exosomes”.

## Data Availability

All data generated or analyzed during this study are included in this published article. The data that support the findings of this study are available from the corresponding author upon request.

## Supplementary Materials

The following supporting information can be downloaded at: https://www.mdpi.com/article/10.3390/ijms231810522/s1.
