# Supplementary material for: Methods of the Large-Scale Production of Extracellular Vesicles
Source: Int J Mol Sci. 2022 Sep 10;23(18):10522. doi: 10.3390/ijms231810522 (PMC9506336; doi:10.3390/ijms231810522)
Supplement: Supplementary file 1 [file ijms-23-10522-s001.zip › ijms-1842421-supplementary.pdf]

Methods of the Large-Scale Production of Extracellular Vesicles

Valeriia Syromiatnikova, Angelina Prokopeva and Marina Gomzikova *

Supplementary materials

Scaling method	Procedure	Links
**Bioreactor culture**		
Multilayer culture vessels	Generation of EVs according to the GMP standards. Cardiac progenitor cells were grown in xeno-free conditions, then 8 L of conditioned medium was harvested with subsequent EVs isolation using TFF.	[20]
Culture plate bioreactor with an attached peristaltic pump and gas delivery system	Cells were subjected to a shear stress by medium flow, that induced higher EVs production compared to static cell culture conditions.	[21]
Bioreactor based on modified flask consisting of two chambers	Two chambers of the flask are separated by a semi-permeable membrane with a molecular weight cut-off 10 kDa. Cells are in one chamber and the medium is in the other. This design eliminates the risk of EVs contamination with serum components.	[22]
Bioreactor combined with microcarriers	Umbilical cord MSCs were cultured in a bioreactor using microcarriers. Then EVs were isolated from a conditioned medium using differential UC or TFF.	[23]
Serum-/xeno-free microcarrier-based culture system in a bioreactor	MSCs were cultured on microcarriers in a Vertical-Wheel bioreactor using a medium supplemented with human platelet lysate.	[24]
Hollow fiber bioreactor	This bioreactor consists of hollow fibers, small semi-permeable membranes with a molecular weight cut-off, which are bound together in a tubular cartridge. The cells are attached to the outside of the hollow fibers, within which the culture medium is continuously pumped, allowing nutrient and gas influx without contamination of EVs with serum components.	[25]
3D-printed scaffold-perfusion bioreactor system	Production of EVs from endothelial cells was significantly increased in a perfusion bioreactor with media circulating at 4 mL/min flow rate.	[27]
Flow stimulation of cells in a bioreactor	Laminar flow of a medium (0.5 or 1.0 mL/min) was applied to stem cells from dental pulp or adipose tissues to induce EVs production.	[28]
**Modulation of cultivation conditions**		
Serum starvation	Restricting cell nutrients by reducing or excluding serum from the medium (use of serum-free medium) led to an increased production of EVs.	[38]
Changing pH	Melanoma cells secreted more EVs in acidic medium.	[41]
Temperature shift	Jurkat cells (human T cell leukemia) and Raji cells (B cells leukemia/lymphoma) were subjected to thermal stress at 40 °C for 1 h, that increased EVs production.	[42]
	Incubation of 3LL lung cancer cells at 42°C for 1 h with subsequent 4 h recovery resulted in increased EVs production.	[44]
Hypoxia	Moderate and severe hypoxia (1% and 0,1% O2, respectively) promote significant release of EVs by breast cancer cells.	[47]
	Ovarian cancer cells, which were exposed to hypoxia, increased EVs production by activation of Rab27a, suppression of Rab7, LAMP1/2, NEU-1 and by stimulation of a more secretory lysosomal phenotype.	[48]
**Chemical induction**		
Sulfhydryl blocking agents	The exposure of 3T3 and SV3T3 mice embryonic cells to a formaldehyde along with a dithiothreitol induce the formation and releasing of plasma membrane vesicles.	[52]
	Sulfhydryl-blocking method was optimized by adding paraformaldehyde with DTT to obtain nanovesicles with high homogeneity.	[54]
	Application of monensin ionophore stimulates the release of EVs from human erythroleukemia cell line K562 in a calcium-dependent manner.	[57]

Agents inducing Ca2+ influx	Ca2+ ionophore treatment was used to increase EVs secretion by oligodendroglial cells, dendritic cells and mast cells.	[58-60]
	Calcium phosphate nanoparticles releasing Ca2+ ions were introduced into murine macrophage-like RAW264.7 cells and human monocyte-like THP-1 cells, which led to a significant increase in EVs secretion.	[61]
Cytochalasins	Cytochalasin B at a concentration of 10 μg/ml promotes the production of vesicles by cells.	[68]
H_2O_2	Oxidative stress stimulates increase in the release of EVs by tumor cells.	[42]
Vesiculation buffer	Obtaining vesicles from highly adherent line of A431 cancer cells based on osmotic shock.	[73]
	Method involves hypotonic washing of cells followed by incubation in osmotic (vesiculating) buffer. The osmotic buffer stressed cells to produce vesicles.	[55]
**Physical stimulation**		
Shear stress	Increasing the duration of shear stress proportionally increases the formation of EVs from the plasma membrane of platelets.	[75]
	Stimulation of fluid shear flow (35 dynes/cm2) increases the production of EVs in osteocyte-like MLO-Y4 cells.	[76]
Acoustic treatment	Exposure of ovarian cancer cells (A2780) to low intensity ultrasound (0.5 W/cm2) for 60 min significantly promotes EVs secretion without significant changes in morphology, size and distribution in vivo.	[78]
	Exposure of cells to high-frequency acoustic radiation (7 cycles, which corresponds to a total treatment duration of 280 min) stimulates EVs generation without affecting cell viability.	[79]
Irradiation	Low-level laser irradiation at a power density of 80 J/cm2 of human endothelial cells leads to increased secretion of EVs.	[80]
	Murine (LL-2) and human (A549) lung cancer cell lines secrete more EVs after exposure to gamma-irradiation (1000 cGy).	[81]
**Production of biomimetic vesicles**		
Homogenization by ultrasound	Cell homogenization by ultrasound leads to rupture and self-assembly of the cell membrane into vesicles.	[82]
Nitrogen cavitation	Nitrogen cavitation rapidly disrupts activated cells to form nanovesicles from the cell membrane.	[83]
Extrusion	Production of EVs-like nanovesicles by disrupting cells after passing through filters with different diameter (10, 5 and 1 μm).	[86]
	Device using centrifugal force and a microsized polycarbonate filter to generate EVs. During centrifugation nanovesicles are produced from cells by elongation the cell membrane while passing through hydrophilic micro-size pores.	[87]
	Generation of artificial nanovesicles via cells flowing through slits in hydrophilic microchannels. Nanovesicles are generated possibly due to abrupt pressure change and their elongated shape caused by shear stress.	[29]
Cutting the cell membrane	Microfluidic system for generating nanovesicles. Cells passing through microchannels are cut by microfabricated 500 nm-thick silicon nitride blades. Afterward, the fragments of plasma membrane, cut from the cells, are self-assembled into spherical nanovesicles.	[88]

EVs – extracellular vesicles
MSCs – mesenchymal stem cells
TFF - tangential flow filtration
UC- ultracentrifugation
DTT - dithiothreitol
